# New Designs of Spectacle Lenses for the Control of Myopia Progression: A Scoping Review

**DOI:** 10.3390/jcm13041157

**Published:** 2024-02-19

**Authors:** Marta Lupon, Carme Nolla, Genis Cardona

**Affiliations:** 1 Vision, Optometry and Health (VOS), Department of Optics and Optometry, Universitat Politècnica de Catalunya, Violinista Vellsolà 37, 08022 Terrassa, Spain; marta.lupon@upc.edu; 2 Terrassa School of Optics and Optometry (FOOT), Universitat Politècnica de Catalunya, Violinista Vellsolà 37, 08022 Terrassa, Spain; carmenely.marcelle.nolla@estudiantat.upc.edu; 3 Applied Optics and Image Processing Group (GOAPI), Department of Optics and Optometry, Universitat Politècnica de Catalunya, Violinista Vellsolà 37, 08022 Terrassa, Spain

**Keywords:** myopia, children, myopia progression, myopia control, spectacle lenses, scoping review

## Abstract

Myopia control with new designs of spectacle lenses is a flourishing area of research. The present work reviews the effectiveness of new designs (DIMSs, defocus-incorporated multiple segments; CARE, cylindrical annular refractive element; HALs/SALs, highly/slightly aspherical lenslets; DOT, diffusion optics technology) aiming at slowing myopia progression. A search through the PubMed database was conducted for articles published between 1 January 2003 and 28 February 2023. Publications were included if they documented baseline central refraction (SER) and/or axial length (AL) data, and the change in these parameters, in myopic children wearing new designs of spectacle lenses (treatment group) compared to myopic children using single-vision lenses, SVLs (control group). The selection process revealed nine suitable articles. Comparing the mean and standard error values of the treatment and control groups, the highest differences in the change in the SER and AL were −0.80 (1.23) D [95% CI: −1.053 to −0.547; *p* < 0.001] and 0.35 (0.05) mm [95% CI: 0.252 to 0.448; *p* < 0.001], respectively; the effect of treatment provided by a HAL design, compared to SVLs, led to a deceleration of 54.8% in the SER and 50.7% in the AL. However, the heterogeneity of the results prevents reaching strong conclusions about the effectiveness of these new designs.

## 1. Introduction

Myopia has a multifactorial etiology, with genetic, environmental, and structural factors playing a critical role [1,2,3]. In recent decades, the prevalence of myopia and high myopia has increased dramatically. In a meta-analysis published in 2016, Holden et al. estimated that between the years 2000 and 2050, the percentage of the global population with myopia would rise from 23% (1406 million people) to 49.8% (4758 million people) [4]. Within the same period, it was predicted that the percentage of high myopia would increase from 2.7% (163 million people) to 9.8% (938 million people). The projected prevalence of myopia for 2020 exceeded 50% in the Asia–Pacific (high income) and East Asia regions and, by 2050, it was predicted that only in certain parts of Africa (Central, East, Southern), Central Asia, and Oceania will this value not be surpassed [4]. This increase in the prevalence of myopia is expected to affect children as well as young and older adults.

Regarding childhood myopia, although the highest prevalence has been reported for countries in East and Southeast Asia, a significant increase has also been observed in other regions. Wang et al. reported a prevalence of myopia of 31.8% in a cross-sectional study of Chinese schoolchildren, with rates of 8.6% in 6-year-olds, increasing to 66.2% and 74.9% in 12-year-olds and 14-year-olds, respectively [5]. Yam et al. observed prevalence rates of 12.7%, 24.4%, and 36.1% in Chinese children aged 6, 7, and 8 years, respectively [6]. Priscilla and Verkicharla described an increase in the prevalence of myopia in India among children aged 5 to 15 years from 4% to 21% between 1999 and 2019, and predicted a rise to 48% by 2050 [7]. In Australia, a follow-up study over 6 years of two cohorts of children aged 12 and 17 reported an increase in myopia prevalence from 1.4% to 14.4% in the younger group, and from 13% to 29.6% in the older group [8]. In Spain, in a recent study, not yet published, an average myopia prevalence of 12.3% was found in a sample of 2489 children of two age groups (6.5% in the 6–7-year-olds and 18.7% in the 11–12-year-olds) [9].

This trend predicts an increase in visual impairment as a consequence of both the rise in uncorrected refractive error and the well-documented association between high myopia and the development of eye conditions such as retinal detachment, glaucoma, myopic maculopathy, or cataracts [10,11]. Moreover, even lower values of myopia are concerning. For instance, in the Australian Blue Mountain Eye Study, 43% of cases of myopic maculopathy presented in individuals with less than −5.00 D (diopter) of myopia, a value commonly considered on the safe side of high myopia [12]. Also, it must be noted that cataract surgery, precise intraocular lens power calculation, and lens implantation are more challenging and entail greater risk when ametropia is high and axial length is beyond the range of normal values [13]. These findings led the authors to suggest that slowing the progression of myopia by only 1.00 D could reduce the likelihood of developing this condition by 40%. Therefore, the alert for myopia progression should not focus solely on high myopia but, as Bullimore and Brenan stated, “each diopter matters” [12].

These findings advocate for urgent measures to reduce the prevalence of myopia and control its progression. Until recently, the primary clinically significant approaches to slowing myopia progression in children were the use of drugs (atropine), special design soft contact lenses, and orthokeratology (Ortho-K). In contrast, evidence regarding the effectiveness of conventional spectacle lenses (single-vision lenses, bifocal lenses, and progressive addition lenses) was inconclusive [14,15,16]. In recent years, however, new designs of spectacle lenses have emerged, which are presented as a hopeful alternative for slowing the progression of myopia. In effect, spectacle lenses avoid the reported side effects of atropine or contact lenses in terms of allergies, potential infections, ocular pain or discomfort, among others; additionally, children can manage this option with a greater level of autonomy.

In short, these new lens designs either create a peripheral myopic retinal defocus or reduce the contrast between adjacent cones, based on the hypothesis that associates the sharpness or contrast of images on the peripheral retina with the onset and progression of myopia [17]. For these purposes, spectacle lens designs may aim at inducing peripheral myopic retinal defocus by combining a single-vision distance zone in the center of the lens surrounded by a more positively powered treatment zone, either with power remaining constant (DIMS, defocus-incorporated multiple-segment design [18]; CARE, cylindrical annular refractive-element design [19]) or changing to some degree (HALs/SALs, highly/slightly aspherical lenslets designs [20,21]; asymmetric progressive horizontal addition [22]), or they may incorporate microscopic diffusers to modulate peripheral contrast (DOT, diffusion optics technology design [23]).

The aim of the present study was to summarize published evidence and compare objective data regarding the effectiveness of these new spectacle lens designs as an alternative strategy for myopia progression control as compared to single-vision lenses (SVLs). Aiming at providing an overall map of the emerging evidence on this topic that might be of interest not only for researchers but also for practitioners (when assessing their patients on myopia progression control options), a scoping review approach was performed [24]. As far as we know, the published literature has not addressed this topic considering this approach. Therefore, the article is structured as a scoping review in accordance with the published PRISMA-ScR guidelines [25].

## 2. Materials and Methods

A literature search was conducted on March 2023 in the PubMed database for articles written in English and published in peer-reviewed journals between 1 January 2003 and 28 February 2023. Using advanced search options, articles were identified by keywords included in the title or abstract. The following search equations were used: [(myopia progression) OR (myopia control) OR (progression of myopia) OR (peripheral defocus)] AND [(spectacle lenses) OR (defocus incorporated multiple segments) OR [(highly OR slightly) AND (aspherical lenslets)]]. A subsequent literature search conducted in the ProQuest database failed to reveal any additional eligible articles.

Eligibility criteria were clinical trials in which the intervention involved at least one treatment group using a new spectacle lens design for the control of myopia progression and a control group with SVLs, and a minimum follow-up period of 6 months. In addition, clinical data regarding the variation in central refraction (spherical equivalent refraction, SER) and/or axial length (AL) for the treatment and control groups needed to be available for inclusion. Studies providing data of peripheral rather than central refraction and those in which the clinical intervention with spectacles consisted only in conventional spectacle lenses (i.e., SVLs, bifocals, or progressive addition lenses) were excluded from the review.

**Figure 1:**
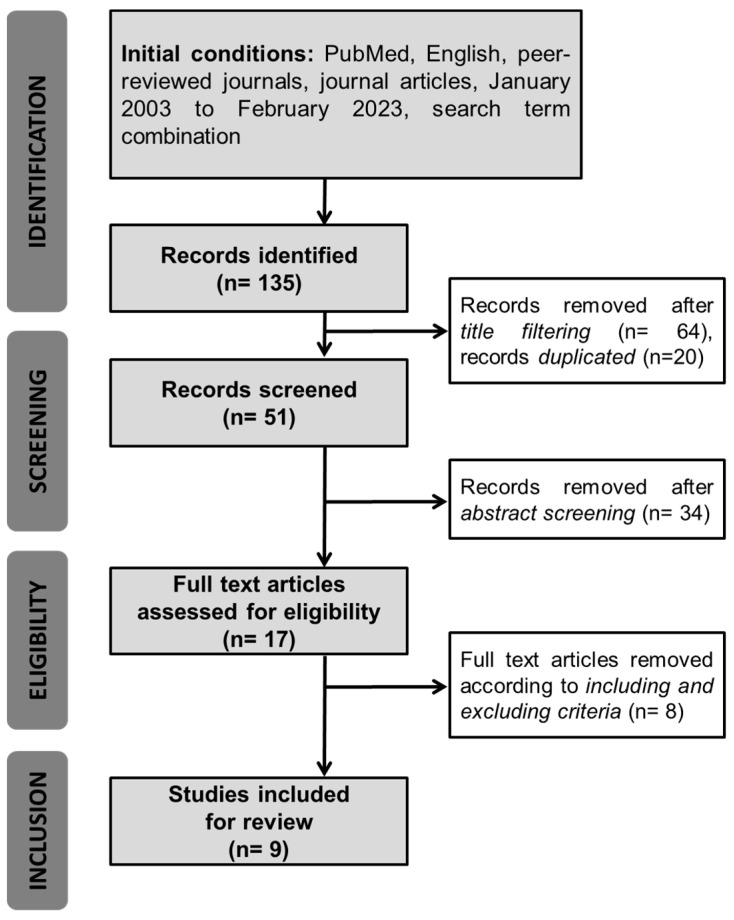
Selection process of the articles for the review.

The literature search query retrieved a total of 135 articles, which were subsequently analyzed by two independent reviewers. After removing duplicate articles (n = 20), the remaining articles were accessed and discarded if the title revealed that the topic of interest was not covered in the corresponding study (n = 64). When the analysis of the title proved inconclusive, the abstract was read considering the eligibility criteria, whereupon an additional 34 articles were excluded. Next, the whole text of the 17 remaining articles was revised, resulting in a further exclusion of 8 articles. Finally, the references of the remaining 9 articles were manually explored to identify studies not retrieved in the original search query, failing to reveal any additional articles (Figure 1).

### Data Analysis

For each of the included articles, the following data were retrieved: baseline SER and/or AL, change in the SER (ΔSER), and/or change in AL (ΔAL) at each follow-up point (relative to baseline). As raw study data were commonly not available, mean and standard deviation (SD) values were used, as provided by the authors (SD was calculated from standard error [SE] and sample size [n] if applicable). With this information, the following parameters were derived:

- Absolute intergroup change: defined as the mean (and SE) difference between the treatment and control groups in the ΔSER and/or ΔAL. The statistical significance of these differences was determined with an independent Student’s *t*-test using the MedCalc Software Ltd. (Ostend, Belgium). Comparison of means calculator (https://www.medcalc.org/calc/comparison_of_means.php, version 22.009; accessed 29 August 2023). The corresponding 95% confidence intervals (95% CI) and *p*-values were obtained, with *p* < 0.05 denoting statistical significance. In terms of the SER, when the absolute intergroup change was a negative number, it denoted that myopia progressed to a greater extent in the control group than in the treatment group, whereas in terms of AL, a positive absolute intergroup change denoted that the AL increased more in the control group than in the treatment group.
- Relative intergroup change (in %): defined as the ratio of the absolute intergroup change to the ΔSER and/or ΔAL of the control group. Negative values of this parameter (referring to both SER and AL) denoted the ratio of myopia progression control due to treatment in terms of the refractive or AL change, meaning its effectiveness (e.g., a value of −50% indicated that the progression of myopia was 50% less in the treatment group than in the control group).
- Relative intragroup change (in %): to determine the progression of the SER and/or AL for the treatment and control group independently, this parameter was defined as the ratio of ΔSER and/or ΔAL to the corresponding baseline values for each group. Positive values denoted the increase in myopia with time (either as the SER or AL changes) for each group.

## 3. Results

Nine articles were included in this scoping review. Although the search period ranged from 1 January 2003 to 28 February 2023, all selected articles were published from 2020 onwards (78% of them in 2022 or 2023). Table 1 presents the complete list of the publications included in the review, with information regarding the following items: the study design, the number of participating children who completed all follow-up appointments, children age range, the total study length and minimum follow-up periods (if applicable), and the type of spectacle lenses dispensed in the study (treatment group).

Most of the included articles describe prospective randomized clinical trials (n = 6, 67%), and the sample ethnicity is predominantly Asian (n = 8, 89% of articles). Considering that the aim of the investigation was to document the effectiveness of new spectacle lens designs compared to SVLs, follow-up data from 6 months to maximum 2 years could be reported (44% of articles followed participants for more than 1 year, n = 4). Spectacle lenses included in the individual studies were the DIMS design (n = 4, 44%) [18,26,27,28], the HAL or SAL designs (3, 33%) [20,29,30], and one study each with the CARE (11%) [19] and DOT (11%) [23] designs.

Whereas all articles provide information on baseline SER and ΔSER for the treatment and control groups, two articles did not collect AL data [27,28]. As reported by the authors, the SER measurements were all conducted under cycloplegic autorefraction, and all AL measurements were obtained with biometers based on optical interference. Some publications collected secondary outcomes, such as visual acuity, phorias, or the presence of adverse effects or discomfort [18,19,20,23,26,29], which are beyond the scope of this review.

**Table 1:** Summary of the articles included in the review.

1st Author, Year (Country)	Study Design	Participants (% Female)	Age Range (Mean ± SD)	Study Length (Follow-Up)	Treatment
Bao [20], 2022 (China)	R/P	161 (54.7)	8–13 years (10.4 ± 1.2)	1 year (6 months)	HALs, SALs
Bao [29], 2022 (China)	R/P	157 (54.1)	8–13 years (10.4 ± 1.2)	2 years (6 months)	HALs, SALs
Huang [26], 2022 (China)	Not R/RS	107 (46.7)	7–12 years (9.1 ± 1.0)	1 year (6 months)	DIMSs, DIMSs + A
Lam [18], 2020 (China)	R/P	160 (43.8)	8–13 years (10.1 ± 1.4)	2 years (6 months)	DIMSs
Liu J [27], 2023 (China)	Not R/RS	10,477 (48.3)	6–16 years (11.0 ± 2.5)	2 years (6 months)	DIMSs
Liu X [19], 2023 (China)	R/P	96 (51.0)	8–12 years (10.0 ± 0.6)	1 year (6 months)	CARE
Long [28], 2023 (China)	Not R/RS	180 (46.1)	6–15 years (10.5 ± 2.1)	1 year (12 months)	DIMSs
Rappon [23], 2021 (China)	R/P	256 (58.2)	6–10 years (8.2 ± 1.4)	1 year (12 months)	DOTa, DOTb
Sankaridurg [30], 2022 (USA)	R/P	119 (45.4)	7–13 years (no data)	1.5 years (6 months)	HALs *

SD, standard deviation; R, randomized; P, prospective; RS, retrospective; HALs, highly aspherical lenslets; SALs, slightly aspherical lenslets; DIMSs, defocus-incorporated multiple segments; A, atropine; CARE, cylindrical annular refractive element; DOT, diffusion optics technology (a and b are two models of DOT lenses, differing in diffusor element density); * data assessed at the 6-month follow-up (subsequent follow-up periods are not applicable due to particularities of the study design).

Table 2 and Table 3 display a summary of the collected information on the baseline SER and ΔSER, and the AL and ΔAL, respectively, as well as the corresponding values of absolute intergroup change, relative intergroup change, and relative intragroup change for these parameters. In some instances, more than one treatment was compared (e.g., Bao [20]: HALs, SALs). When data were provided for several follow-up points, it was presented independently.

Statistically significant differences in the SER between groups (absolute intergroup change) at the 2-year follow-up points were evidenced in all studies including this follow-up period, with a maximum value of −0.80 D, obtained with HAL lenses [29]. However, the best rate of myopia progression control (relative intergroup change) in this follow-up point corresponded to a DIMS design (59.14%) [18]. Myopia control was not found to be effective in terms of the SER in two of the five studies including a 6-month follow-up period (CARE design [19], HAL design [30]), and with the CARE design at the 1-year follow-up point [19]. Studies reporting a positive effect of the treatment presented absolute intergroup change values ranging from −0.17 D to −0.24 D at 6 months, from −0.27 D to −0.54 D at 1 year, and from −0.25 D to −0.80 D at 2 years.

In terms of the AL, all studies reporting a follow-up of 1 or 2 years found statistically significant differences between groups (absolute intergroup change), with a maximum difference in 0.35 mm with the HAL design [29]. As occurred with the SER, the best rate of ocular elongation control (relative intergroup change) was also obtained with the DIMS design (60.38%) [18]. At the 6-month follow-up, two out of five studies did not report a positive effect of the treatment on the AL (CARE design [19], SAL design [20]). Absolute intergroup change values of the studies observing a significant effect on the AL ranged from 0.07 to 0.17 mm at 6 months, from 0.11 to 0.23 mm at 1 year, and from 0.18 to 0.35 mm at 2 years.

## 4. Discussion

The purpose of the present review was to summarize published research on the effectiveness of spectacle lenses with new designs for myopia control as compared to single-vision lenses in terms of the changes in the central refraction and axial length. In published clinical studies, the methods employed by researchers to determine the effectiveness of a particular lens design are often not fully described and remain heterogeneous. Aiming at clarity and uniformity of presentation, three new parameters were introduced, namely, absolute intergroup change, relative intergroup change, and relative intragroup change.

Previous research on myopia control strategies has mainly focused on atropine, soft contact lenses, and Ortho-K [31,32], and only a few spectacle lens designs have been included, given their recent development, which precludes the long-term follow-up of their effectiveness. Indeed, 78% of the included studies in this review were published in the last 14 months, thus suggesting that this is a relatively novel and fertile field of research.

The present findings revealed that, whereas at 6 months, the differences between new spectacle designs and SVLs were not always significant, this trend was reversed at the 1-year and 2-year follow-ups both for the SER and AL. This was particularly manifest with the DIMS and HAL designs, in which differences between 50% and 60% were found in these parameters. However, the results reported by the authors of the various studies included in this review were not homogeneous, in agreement with a previous systematic review comparing atropine, soft contact lenses, Ortho-K, and the DIMS and HAL spectacle lens designs, which documented a high variability in the performance of the DIMS and HAL designs [32].

This scoping review only included articles comparing spectacle lens designs for myopia control with SVLs. Previous research is available in which the control group used another myopia control strategy. Thus, Du et al. compared the HAL design with a combined treatment of SVLs and atropine and with Ortho-K. At the 1-year follow-up, the SER increased 23.1% with HALs, 8.6% with SVLs and atropine, which the authors attributed to the effect of atropine, and 13.6% with Ortho-K [33]. One of the articles included in this review also found an increased effect of a combined treatment with DIMSs and atropine versus DIMSs alone (54% versus 26% in the SER, and 46% versus 21% in AL) [26]. In turn, Guo et al. compared two myopia control strategies with spectacle lenses, namely, the DIMS and HAL designs [34]. At the 1-year follow-up, they encountered better results with the HAL design, with an absolute intergroup change of −0.29 D [95% CI: −0.44 to −0.13; *p* < 0.001] in the SER and 0.11 mm [95% CI: 0.02 to 0.20; *p* = 0.03] in the AL.

Other longer-term studies in which the follow-up extended beyond the 2-year period did not compare myopia control designs with SVLs after this point. For instance, Lam and coworkers continued their initial research (described above in this review, [18]) to include 3-year [35] and 6-year follow-ups [36], albeit the control group who had been using SVLs up to the 2-year follow-up changed to DIMSs afterwards. Similarly, Bao and coworkers continued their initial study [29] for one more year, but from the two-year follow-up onwards, all participants (including the previous control group with SVLs) wore the HAL design [37]. Finally, the research of Sankaridurg and coworkers consisted in a first phase (6 months) in which the HAL design was compared with a control group using SVLs, included in this review, and a second 12-month-long phase in which the control group also used the HAL design [30]. Results from this second phase are not addressed in this review.

It must also be noted that, to be eligible, articles needed to provide complete information on at least the SER (baseline and changes over time), and, if available, the same data on the AL. However, two of the included articles (out of nine) failed to report changes in the AL [27,28]. The initial literature research revealed at least two more studies comparing new spectacle lens designs for myopia control with SVLs, but data incompleteness prevented their inclusion in this review [38,39].

Finally, it is important to highlight that, out of the nine articles, only in one of them was the sample of non-Asian ethnicity. Therefore, the scarcity of data in the non-Asian population does not permit us to determine whether ethnicity may be a factor to consider when evaluating the effectiveness of the new designs of spectacle lenses for myopia progression control.

This study is not devoid of limitations, mainly arising from the availability of the original eligible resources. Thus, the analysis was limited to summary outcomes (mean and SD, or SE), as raw data were generally not provided by the authors. However, the specific indications for conducting a scoping review [24] were considered optimal to present inclusive results describing a general overview of all available evidence. Additionally, the heterogeneity in study designs, follow-up periods, lens designs, data presentations, and other aspects, determined the format of this scoping review over a more robust investigation, such as a meta-analysis, which is considered to represent the highest level of scientific evidence. Also, research on these spectacle lens designs of recent development is, by definition, subject to time constraints, particularly manifest in the short follow-up periods (6 months) of some of the included articles. It may be assumed that new evidence arising from studies with a longer follow-up shall be published in the near future. In this regard, given the relative novelty of this topic of research, practitioners with an interest in myopia control strategies are strongly recommended to keep up-to-date with the most recent publications.

## 5. Conclusions

This scoping review, which included a limited number of eligible articles comparing new spectacle lens designs for myopia control with SVLs, revealed that some of these designs proved effective to control myopia progression in children. Albeit further research with longer follow-up periods is needed to allow for sounder conclusions, the potential advantage of using these new spectacle lens designs over other options, such as atropine instillation, soft contact lenses, or Ortho-K, should be considered within the overall framework of aspects such as safety, comfort, cost, and child autonomy. This information should be very relevant to practitioners when advising concerned parents of children with myopia on the best alternatives for myopia control.

## Figures and Tables

**Table 2:** SER analysis of the included articles. When multiple follow-ups were conducted within the same study, the data are presented based on each follow-up period.

Authors	Follow-Up (m, Months)	TX (n)	SVL (n)	Baseline SER (Mean ± SD) D		ΔSER at Follow-Up (Mean ± SD) D		Absolute Intergroup Change Mean (SE), [95% CI] D		Relative Intergroup Change	Relative Intragroup Change	
				TX	SVL	TX	SVL		*p*		TX	SVL
Bao [20]	6 m	HALs (54)	(52)	−2.70 ± 1.03	−2.46 ± 0.87	−0.10 ± 0.29	−0.34 ± 0.29	−0.24 (0.06), [−0.35 to −0.13]	<0.001 *	−70.59%	3.70%	13.82%
		SALs (55)		−2.31 ± 0.96		−0.17 ± 0.30		−0.17 (0.06), [−0.28 to −0.06]	0.004 *	−50.00%	7.36%	
Lam [18]	6 m	DIMSs (79)	(81)	−2.97 ± 0.97	−2.76 ± 0.96	−0.13 ± 0.27	−0.37 ± 0.36	−0.24 (0.05), [−0.34 to −0.14]	<0.001 *	−64.86%	4.38%	13.41%
Liu X [19]	6 m	CARE (52)	(44)	−2.67 ± 0.69	−2.56 ± 0.75	−0.38 ± 0.35	−0.47 ± 0.37	−0.09 (0.07), [−0.24 to 0.06]	0.224	−19.15%	14.23%	18.36%
Sankaridurg [30]	6 m	HALs (54)	(65)	−3.47 ± 1.16	−3.37 ± 1.22	−0.20 ± 0.31	−0.27 ± 0.42	−0.07 (0.07), [−0.21 to 0.07]	0.312	−25.93%	5.76%	8.01%
Bao [20]	1 year	HALs (54)	(52)	−2.70 ± 1.03	−2.46 ± 0.87	−0.27 ± 0.44	−0.81 ± 0.43	−0.54 (0.08), [−0.71 to −0.37]	<0.001 *	−66.67%	10.00%	32.93%
		SALs (55)		−2.31 ± 0.96		−0.48 ± 0.37		−0.33 (0.08), [−0.48 to −0.18]	<0.001 *	−40.74%	20.78%	
Huang [26]	1 year	DIMSs (30)	(38)	−2.84 ± 1.24	−2.60 ± 0.99	−0.79 ± 0.47	−1.07 ± 0.64	−0.28 (0.14), [−0.56 to −0.00]	0.049 *	−26.17%	27.82%	41.15%
Lam [18]	1 year	DIMSs (79)	(81)	−2.97 ± 0.97	−2.76 ± 0.96	−0.17 ± 0.44	−0.55 ± 0.36	−0.38 (0.06), [−0.51 to −0.25]	<0.001 *	−69.09%	5.72%	19.93%
Liu X [19]	1 year	CARE (52)	(44)	−2.67 ± 0.69	−2.56 ± 0.75	−0.56 ± 0.46	−0.71 ± 0.39	−0.15 (0.09), [−0.32 to 0.02]	0.091	−21.13%	20.97%	27.73%
Liu J [27]	1 year	DIMSs (2240)	(2240)	−2.88 ± 1.74	−2.85 ± 1.87	−0.50 ± 0.43	−0.77 ± 0.58	−0.27 (0.01), [−0.30 to −0.24]	<0.001 *	−35.06%	17.36%	27.02%
Long [28]	1 year	DIMSs (90)	(90)	−3.82 ± 1.57	−3.75 ± 1.51	−0.51 ± 0.50	−0.85 ± 0.51	−0.34 (0.07), [−0.49 to −0.19]	<0.001 *	−40.00%	13.35%	22.67%
Rappon [23]	1 year	DOTa (88)	(93)	−2.00 ± 0.93	−1.95 ± 1.02	−0.15 ± 0.39	−0.53 ± 0.46	−0.38 (0.06), [−0.51 to −0.25]	<0.001 *	−71.70%	7.50%	27.18%
		DOTb (75)		−1.85 ± 0.91		−0.23 ± 0.49		−0.30 (0.07), [−0.45 to −0.15]	0.001 *	−56.60%	12.43%	
Bao [29]	2 years	HALs (54)	(50)	−2.70 ± 1.03	−2.44 ± 0.85	−0.66 ± 0.66	−1.46 ± 0.64	−0.80 (0.13), [−1.05 to −0.55]	0.001 *	−54.79%	24.44%	59.84%
		SALs (53)		−2.28 ± 0.95		−1.04 ± 0.44		−0.42 (0.11), [−0.63 to −0.21]	0.002 *	−28.77%	45.61%	
Lam [18]	2 years	DIMSs (79)	(81)	−2.97 ± 0.97	−2.76 ± 0.96	−0.38 ± 0.53	−0.93 ± 0.54	−0.55 (0.08), [−0.72 to −0.38]	<0.001 *	−59.14%	12.79%	33.70%
Liu J [27]	2 years	DIMSs (735)	(735)	−3.00 ± 1.64	−2.92 ± 1.94	−0.88 ± 0.62	−1.23 ± 0.76	−0.35 (0.04), [−0.42 to −0.28]	<0.001 *	−28.46%	29.33%	42.12%
		DIMSs (234)	(234)	−2.92 ± 1.54	−2.93 ± 1.99	−0.91 ± 0.45	−1.16 ± 0.45	−0.25 (0.04), [−0.33 to −0.17]	<0.001 *	−21.55%	31.16%	39.59%

TX, treatment group; SVL, single-vision lenses group; D, diopter; SER, spherical equivalent refraction; SD, standard deviation; SE, standard error; HALs, highly aspherical lenslets; SALs, slightly aspherical lenslets; DIMSs, defocus-incorporated multiple segments; CARE, cylindrical annular refractive element; DOT, diffusion optics technology (a and b are two models of DOT lenses, differing in diffusor element density); * denotes statistical significance.

**Table 3:** AL analysis of the included articles. When multiple follow-ups were conducted within the same study, the data were presented based on each follow-up period.

Authors	Follow-Up (m, Months)	TX (n)	SVL (n)	Baseline AL (Mean ± SD) mm		ΔAL at Follow-Up (Mean ± SD) mm		Absolute Intergroup Change Mean (SE), [95% CI] mm		Relative Intergroup Change	Relative Intragroup Change	
				TX	SVL	TX	SVL		*p*		TX	SVL
Bao [20]	6 m	HALs (54)	(52)	24.76 ± 0.66	24.77 ± 0.65	0.08 ± 0.07	0.20 ± 0.07	0.12 (0.01), [0.09 to 0.15]	<0.001 *	−60.00%	0.32%	0.81%
		SALs (55)		24.43 ± 0.74		0.14 ± 0.07		0.06 (0.10), [0.13 to 0.25]	0.540	−30.00%	0.57%	
Lam [18]	6 m	DIMSs (79)	(81)	24.70 ± 0.82	24.60 ± 0.83	0.03 ± 0.09	0.20 ± 0.09	0.17 (0.01), [0.14 to 0.20]	<0.001 *	−85.00%	0.12%	0.81%
Liu X [19]	6 m	CARE (52)	(44)	24.65 ± 0.67	24.66 ± 0.63	0.19 ± 0.12	0.23 ± 0.12	0.04 (0.03), [0.01 to 0.02]	0.107	−17.39%	0.77%	0.93%
Sankaridurg [30]	6 m	HALs (54)	(65)	25.10 ± 0.80	24.90 ± 0.80	0.06 ± 0.15	0.13 ± 0.15	0.07 (0.03), [0.01 to 0.12]	0.013 *	−53.85%	0.24%	0.52%
Bao [20]	1 year	HALs (54)	(52)	24.76 ± 0.66	24.77 ± 0.65	0.13 ± 0.15	0.36 ± 0.14	0.23 (0.03), [0.17 to 0.29]	<0.001 *	−63.89%	0.53%	1.45%
		SALs (55)		24.43 ± 0.74		0.25 ± 0.15		0.11 (0.03), [0.05 to 0.17]	0.002 *	−30.56%	1.02%	
Huang [26]	1 year	DIMSs (30)	(38)	24.62 ± 0.87	24.60 ± 0.71	0.41 ± 0.22	0.52 ± 0.22	0.11 (0.05), [0.00 to 0.22]	0.045 *	−21.15%	1.67%	2.11%
Lam [18]	1 year	DIMSs (79)	(81)	24.70 ± 0.82	24.60 ± 0.83	0.11 ± 0.18	0.32 ± 0.18	0.21 (0.03), [0.15 to 0.27]	<0.001 *	−65.63%	0.45%	1.30%
Liu X [19]	1 year	CARE (52)	(44)	24.65 ± 0.67	24.66 ± 0.63	0.26 ± 0.18	0.36 ± 0.16	0.10 (0.03), [0.03 to 0.17]	0.005 *	−27.78%	1.05%	1.46%
Liu J [27]	1 year	DIMSs (2240)	(2240)	-	-	-	-	*No data of AL*	-	-	-	-
Long [28]	1 year	DIMSs (90)	(90)	-	-	-	-	*No data of AL*	-	-	-	-
Rappon [23]	1 year	DOTa (88)	(93)	24.09 ± 0.82	24.03 ± 0.70	0.15 ± 0.15	0.30 ± 0.17	0.15 (0.02), [0.10 to 0.20]	<0.001 *	−50.00%	0.62%	1.25%
		DOTb (75)		23.94 ± 0.70		0.18 ± 0.21		0.12 (0.03), [0.06 to 0.18]	0.001 *	−40.00%	0.75%	
Bao [29]	2 years	HALs (54)	(50)	24.76 ± 0.66	24.77 ± 0.64	0.34 ± 0.22	0.69 ± 0.28	0.35 (0.05), [0.25 to 0.45]	<0.001 *	−50.72%	1.37%	2.79%
		SALs (53)		24.44 ± 0.73		0.51 ± 0.29		0.18 (0.06), [0.07 to 0.29]	0.002 *	−26.09%	2.09%	
Lam [18]	2 years	DIMSs (79)	(81)	24.70 ± 0.82	24.60 ± 0.83	0.21 ± 0.18	0.53 ± 0.27	0.32 (0.04), [0.25 to 0.39]	<0.001 *	−60.38%	0.85%	2.15%
Liu J [27]	2 years	DIMSs (735)	(735)	-	-	-	-	*No data of AL*	-	-	-	-
		DIMSs (234)	(234)	-	-	-	-	*No data of AL*	-	-	-	-

TX, treatment group; SVL, single-vision lenses group; AL, axial length; SD, standard deviation; SE, standard error; HALs, highly aspherical lenslets; SALs, slightly aspherical lenslets; DIMSs, defocus-incorporated multiple segments; CARE, cylindrical annular refractive element; DOT, diffusion optics technology (a and b are two models of DOT lenses, differing in diffusor element density); * denotes statistical significance.

## Data Availability

Data may be available upon reasonable request to the authors.
